# Associations between reflux esophagitis and the progression of coronary artery calcification: A cohort study

**DOI:** 10.1371/journal.pone.0184996

**Published:** 2017-10-05

**Authors:** Yang Won Min, Byeong Geun Song, Hye Seung Kim, Kyunga Kim, Hyuk Lee, Byung-Hoon Min, Jun Haeng Lee, Hee Jung Son, Poong-Lyul Rhee, Jae J. Kim

**Affiliations:** 1 Department of Medicine, Samsung Medical Center, Sungkyunkwan University School of Medicine, Seoul, South Korea; 2 Biostatistics and Clinical Epidemiology Center, Samsung Medical Center, Seoul, South Korea; 3 Center for Health Promotion, Samsung Medical Center, Seoul, South Korea; Nagoya University, JAPAN

## Abstract

**Background:**

Reflux esophagitis (RE) and coronary heart disease (CHD) have common risk factors, including obesity and metabolic syndrome. This study aimed to evaluate the associations between RE and the future CHD risk.

**Methods:**

This retrospective cohort study included 8,221 participants who were ≥20 years old, and who underwent esophagogastroduodenoscopy and coronary computed tomography (CT) scans during the same visit and subsequent CT scans between 2003 and 2013. RE was defined as the presence of at least Los Angeles classification grade A mucosal break. CT scan was used to determine the coronary artery calcium (CAC) scores. CAC progression was defined as an increase in the CAC score on a subsequent CT scan.

**Results:**

RE was present in 984 (12.0%) participants. RE at baseline was associated with CAC progression (odds ratio [OR], 1.253; 95% confidence interval [CI], 1.088–1.444; *P* = 0.002), and this association persisted after adjusting the model for age, sex, smoking status, and alcohol consumption (OR, 1.175; 95% CI, 1.001–1.378; *P* = 0.048). This association disappeared when the model was further adjusted for body mass index, diastolic blood pressure, the presence of hypertension, glycated hemoglobin, low-density lipoprotein cholesterol, and triglycerides (OR, 1.088; 95% CI, 0.924–1.281; *P* = 0.311) which were selected using a stepwise selection procedure from several metabolic variables.

**Conclusions:**

Our results suggest that the presence of RE is closely associated with CHD, even though RE is not a direct risk factor for CHD. Metabolic factors may play roles in CAC progression in individuals with RE.

## Introduction

Gastroesophageal reflux disease (GERD) is widespread and its global burden is increasing. The GERD prevalence estimates are 18.1%–27.8% in North America, 8.8%–25.9% in Europe, and 2.5%–7.8% in East Asia [1,2]. Evidence suggests that the prevalence of GERD has increased, particularly in North America and East Asia [1]. The most important factor associated with the development of GERD is central obesity [3–6]. Given that obesity is also an important determinant of coronary heart disease (CHD), it is reasonable to assume that GERD may reflect the risk of CHD [7]. Furthermore, GERD and CHD share metabolic syndrome as a common risk factor. However, no data are available that describe the association between GERD and CHD.

The coronary artery calcium (CAC) score is an established marker of subclinical atherosclerosis, and it is an independent predictor of CHD events in the asymptomatic population [8,9]. Furthermore, the progression of the CAC score is closely associated with an increased risk of CHD events, and it even predicts all-cause mortality [10,11]. Hence, CAC scores are widely used to evaluate the risk of CHD. Thus, this study aimed to assess the associations between GERD and the future risk of CHD, which were determined using CAC scoring, in a large cohort of men and women who participated in a health screening program.

## Patients and methods

### Study design and patient population

**Fig 1:**
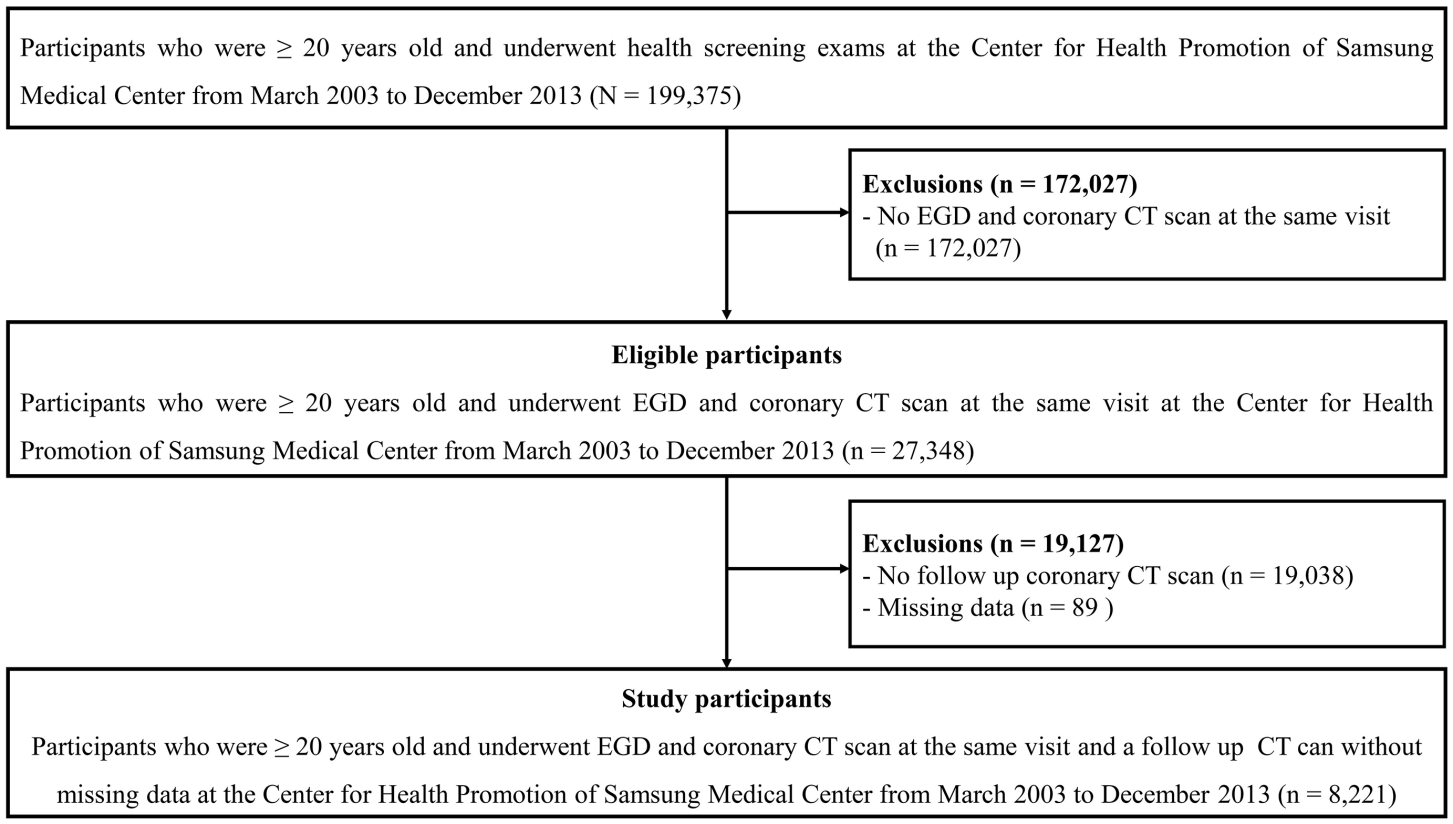
Study flow chart. EGD, esophagogastroduodenoscopy; CT, computed tomography.

This cohort study included 199,375 men and women who were aged ≥20 years and underwent health screening examinations at the Health Promotion Center of the Samsung Medical Center in Seoul, South Korea, from March 1, 2003 to December 31, 2013. Esophagogastroduodenoscopy (EGD) was conducted in all participants since Korea has the high incidence of gastric cancer, and CAC scan was conducted only if the participants wanted to do regardless of cardiovascular risk factor. Given that our objective was to prospectively evaluate the associations between reflux esophagitis (RE) and the changes in the CAC score, only participants who underwent screening EGD and coronary computed tomography (CT) scanning during the same visit were enrolled (*n* = 27,348). We then excluded participants who did not undergo follow-up coronary CT scans (*n* = 19,038) and those who had missing information (*n* = 89). Finally, a total of 8,221 participants who were aged ≥20 years, had undergone EGD and repeated coronary CT scans, and who did not have any data missing were included in the analysis (Fig 1).

This study protocol was conducted in accordance with the Declaration of Helsinki. The Institutional Review Board of the Samsung Medical Center approved the study’s protocol and waived the requirement for informed consent, because we used de-identified data that were routinely collected during the health screening visits.

### Data collection

Data describing the participants’ demographics, smoking status, alcohol consumption, and past medical histories were collected using standardized, self-administered questionnaires. Hypertension was defined as a systolic blood pressure (SBP) ≥140 mmHg, a diastolic blood pressure (DBP) ≥90 mmHg, a self-reported history of hypertension, or the current use of antihypertensive medications. Diabetes mellitus was defined as fasting serum glucose ≥126 mg/dL, a self-reported history of diabetes, or the self-reported use of insulin or antidiabetic medications. Dyslipidemia was defined as high-density lipoprotein (HDL) cholesterol <40 mg/dL in men, HDL cholesterol <50 mg/dL in women, triglycerides ≥150 mg/dL, or the use of medication for dyslipidemia. Information about the presence of ischemic heart disease and cerebrovascular disease was collected from the participants’ medical records. A participant’s smoking status was categorized as never, past, or current smoker. A participant’s alcohol consumption was categorized as never, past, or current drinker. Trained nurses measured the body mass index (BMI) in kg/m^2^, waist circumference (WC) in cm, SBP, and DBP. The glycated hemoglobin (HbA1c), total cholesterol, low-density lipoprotein cholesterol, HDL cholesterol, and triglyceride levels were also measured.

Since there is no gold standard for a diagnosis of GERD, a definition of GERD is usually based on the presence of reflux symptoms. In our study, however, we used the presence of RE as an objective marker of GERD to reduce variability related to symptom reporting. RE was defined as the presence of at least a grade A mucosal break that was detected during EGD, which was based on the Los Angeles classification of esophagitis [12].

### Coronary computed tomography

Brilliance 40 (Philips Medical Systems, Cleveland, Ohio), VCT LightSpeed 64 (GE Healthcare, Milwaukee, Wisconsin), or Discovery 750HD (GE Healthcare) multidetector CT scanners were used to acquire images of CAC. The scans were analyzed using Extended Brilliance Workspace (Philips Medical Systems) or Advantage (GE Healthcare) workstations. The CAC scores were calculated as described by Agatston *et al*. [13].

### Statistical analysis

The patients’ baseline characteristics were compared according to the RE status using the chi-square test or Student’s t-test. CAC progression was defined as an increase in the CAC score on a subsequent CT scan. To estimate the risk of CAC progression according to the RE status at baseline, odds ratios (ORs) and 95% confidence intervals (CIs) were calculated using logistic regression analysis which deals with CAC changes as a binary variable (increase or not). We further estimated the ORs and 95% CIs using adjusted models, as follows: model 1 was adjusted for age and sex; model 2 was further adjusted for alcohol consumption and the smoking status; and model 3 was further adjusted for BMI, DBP, the presence of hypertension, HbA1c, LDL cholesterol, and triglycerides (log_e_ transformed), which were selected using a stepwise selection procedure from several metabolic variables, including BMI, height, weight, SBP, DBP, HbA1c, total cholesterol, LDL cholesterol, HDL cholesterol, and triglycerides, and the presence of hypertension, diabetes, dyslipidemia, ischemic heart disease, and cerebrovascular disease. The data were screened for multicollinearity.

All of the *P* values reported are two-sided, and *P* values <0.05 were considered statistically significant. SAS version 9.4 (SAS Institute, Cary, NC) was used for the statistical analyses.

## Results

The baseline cross sectional analysis (n = 27,348) of those with RE and extent of CAC controlling other factors are shown in Table 1. We included 8,211 participants in the analysis, comprising 7,237 participants who did not have RE and 984 participants who had RE. The study participants’ baseline characteristics are shown in Table 2. The participants’ mean age was 53.8±7.7 years, and 12.0% of the participants had RE at baseline (*n* = 984). Compared to the participants who did not have RE, those who had RE were more likely to be men, current smokers, and current drinkers, and they were more likely to have higher BMIs, DBPs, and triglyceride levels, and lower HDL cholesterol levels, and to be taller and heavier. At baseline, the mean CAC score was higher in the participants who had RE (99.6±276.4) than in those who did not have RE (76.6±202.6) (*P* = 0.012). The participants who had RE at baseline had a significantly higher CAC progression rate (67.2%) than those who did not have RE at baseline (62.0%) (*P* = 0.006).

**Table 1:** Cross sectional univariate and multivariate analyses of the association between reflux esophagitis and coronary artery calcium score.

Variables	Univariate analysis		Multivariate analysis	
	OR (95% CI)	*P* value	OR (95% CI)	*P* value
Reflux esophagitis	1.316 (1.221–1.419)	<0.001	1.199 (1.100–1.307)	<0.001
BMI (kg/m^2^)	1.090 (1.080–1.100)	<0.001	1.090 (1.070–1.111)	<0.001
Height (cm)	1.000 (0.997–1.003)	0.930		
Weight (kg)	1.016 (1.014–1.019)	<0.001	0.986 (0.981–0.991)	<0.001
Smoking status		<0.001		
Never	1		1	
Past smoker	1.695 (1.592–1.804)	<0.001	1.738 (1.618–1.866)	<0.001
Current smoker	1.265 (1.184–1.351)	<0.001	1.252 (1.159–1.352)	<0.001
Alcohol consumption		<0.001		
Never	1		1	
Past drinker	1.667 (1.282–2.167)	<0.001	1.434 (1.082–1.901)	0.012
Current drinker	1.060 (0.997–1.128)	<0.062	0.994 (0.922–1.072)	0.871
SBP (mmHg)	1.015 (1.014–1.017)	<0.001	1.015 (1.012–1.018)	<0.001
DBP (mmHg)	1.014 (1.012–1.016)	<0.001	0.992 (0.988–0.995)	<0.001
HbA1c (%)	1.667 (1.605–1.732)	<0.001	1.571 (1.503–1.642)	<0.001
Total cholesterol (mg/dL)	1.000 (1.000–1.001)	0.391		
LDL-C (mg/dL)	1.001 (1.000–1.002)	0.024	1.001 (1.000–1.002)	0.020
HDL-C (mg/dL)	0.988 (0.987–0.990)	<0.001	0.993 (0.990–0.995)	<0.001
Triglycerides (mg/dL)^1^	1.001 (1.001–1.002)	<0.001	1.000 (1.000–1.000)	0.819
Hypertension	1.784 (1.633–1.950)	<0.001	1.415 (1.263–1.585)	<0.001
Diabetes	2.163 (1.871–2.500)	<0.001	0.910 (0.754–1.098)	0.323
Dyslipidemia	1.296 (1.173–1.432)	<0.001	0.935 (0.824–1.061)	0.298
Ischemic heart disease	3.070 (2.121–4.444)	<0.001	1.952 (1.259–3.026)	0.003
Cerebrovascular disease	1.985 (1.116–3.530)	0.020	0.828 (0.391–1.752)	0.622

OR, odds ratio; CI, confidence interval; BMI, body mass index; SBP, systolic blood pressure; DBP, diastolic blood pressure; LDL-C, low-density lipoprotein cholesterol; HDL-C, high-density lipoprotein cholesterol.

^1^log_e_ transformed.

**Table 2:** Baseline characteristics of the study participants according to the reflux esophagitis status.

	Overall (n = 8,221)	Reflux esophagitis status		p value
		Negative (n = 7,237)	Positive (n = 984)	
Age (years)	53.8±7.7	53.8±7.7	53.8±7.8	0.891
Male sex	7,481 (91)	6,516 (90)	965 (98.1)	<0.001
BMI (kg/m^2^)	24.7±2.6	24.6±2.6	25.4±2.4	<0.001
Height (cm)	169.4±6.7	169.3±6.8	170.4±5.9	<0.001
Weight (kg)	71.1±9.5	70.8±9.5	73.7±8.6	<0.001
Smoking status				<0.001
Never	2,221 (30.6)	2,062 (32.2)	159 (18.6)	
Past smoker	3,295 (45.4)	2,918 (45.6)	377 (44.1)	
Current smoker	1,740 (24.0)	1,421 (22.2)	319 (37.3)	
Alcohol consumption				<0.001
Never	1,332 (17.0)	1,231 (17.9)	101 (10.7)	
Past drinker	26 (0.3)	24 (0.4)	2 (0.2)	
Current drinker	6,466 (82.6)	5,626 (81.8)	840 (89.1)	
CAC score >0	4,932 (60.0)	4,302 (59.4)	630 (64.0)	0.006
CAC difference (final—baseline) >0	5,149 (62.6)	4,488 (62.0)	661 (67.2)	0.002
CAC score (Agatston units)	79.3±212.9	76.6±202.6	99.6±276.4	0.012
SBP (mmHg)	119.4±15.6	119.3±15.6	120.2±15.4	0.065
DBP (mmHg)	75.7±10.5	75.5±10.5	77.0±10.5	<0.001
HbA1c (%)	5.64±0.8	5.63±0.7	5.68±0.9	0.246
Total cholesterol (mg/dL)	198.6±34.2	198.7±34.1	197.9±35.0	0.873
LDL-C (mg/dL)	128.4±31.0	128.6±30.9	126.8±31.6	0.253
HDL-C (mg/dL)	51.8±12.7	52.1±12.7	49.8±12.0	<0.001
Triglycerides (mg/dL)	147.2±88.1	145.0±86.2	163.0±99.6	<0.001
Hypertension	631 (7.7)	553 (7.6)	78 (7.9)	0.752
Diabetes	239 (2.9)	210 (2.9)	29 (3.0)	0.937
Dyslipidemia	538 (6.5)	485 (6.7)	53 (5.4)	0.118
Ischemic heart disease	28 (0.3)	26 (0.4)	2 (0.2)	0.570
Cerebrovascular disease	8 (0.1)	5 (0.1)	3 (0.3)	0.060

BMI, body mass index; CAC, coronary artery calcium; SBP, systolic blood pressure; DBP, diastolic blood pressure; HbA1c, glycated hemoglobin; LDL-C, low-density lipoprotein cholesterol; HDL-C, high-density lipoprotein cholesterol. The data presented are the means±standard deviations or numbers (percentages).

The CAC scores increased in the 4,488 participants who did not have RE at baseline during 28,169.16 person-years of follow-up, and the incidence was 159.3/1,000 person-years. The CAC scores increased in the 661 participants who had RE at baseline during 3,723.78 person-years of follow-up, and the incidence was 177.5/1,000 person-years. A comparison of the participants who had RE with those who did not have RE in the univariate analysis showed that the OR for CAC progression was 1.253 (95% CI, 1.088–1.444; *P* = 0.002). After adjusting the model for age and sex, the OR for CAC progression was 1.227 (95% CI, 1.058–1.423; *P* = 0.007). When the model was further adjusted for alcohol consumption and the smoking status and the participants with RE were compared with those who did not have RE, the OR for CAC progression was 1.175 (95% CI 1.001–1.378; *P* = 0.048). This association disappeared in model 3 that was further adjusted for BMI, DBP, the presence of hypertension, HbA1c, LDL cholesterol, and triglycerides (OR, 1.088; 95% CI, 0.924–1.281; *P* = 0.311) (Table 3). The results from the multivariate analysis are shown in Table 4.

**Table 3:** Ratio of the coronary artery calcium (CAC) score progression according to the reflux esophagitis status at baseline.

	Reflux esophagitis status		*P* value
	Negative (n = 7,237)	Positive (n = 984)	
Mean±SD follow-up duration (years)	3.9±2.2	3.8±2.1	0.178
Person-years	28,169.2	3,723.8	
Incident cases of CAC progression	4,488	661	
Incidence rate per 1,000 person-years	159.3	177.5	
CAC progression ratio			
Crude	1 (reference)	1.253 (1.088–1.444)	0.002
Model 1	1 (reference)	1.227 (1.058–1.423)	0.007
Model 2	1 (reference)	1.175 (1.001–1.378)	0.048
Model 3	1 (reference)	1.088 (0.924–1.281)	0.311

SD, standard deviation; CAC, coronary artery calcium. Values in parenthesis are the 95% confidence intervals. Model 1: Adjusted for age and sex. Model 2: Further adjusted for baseline smoking status (never, past, or current), and alcohol consumption (never, past, or current). Model 3: Further adjusted for body mass index, diastolic blood pressure, hypertension, glycated hemoglobin, low-density lipoprotein-cholesterol, and triglycerides (log_e_-transformed).

**Table 4:** Univariate and multivariate analyses of the association between reflux esophagitis and coronary artery calcium score progression.

Variables	Univariate analysis		Multivariate analysis	
	OR (95% CI)	*P* value	OR (95% CI)	*P* value
Reflux esophagitis	1.253 (1.088–1.444)	0.002	1.088 (0.924–1.281)	0.311
Age (years)	1.091 (1.084–1.099)	<0.001	1.101 (1.092–1.111)	<0.001
Sex (male)	1.165 (0.999–1.359)	0.051	1.442 (1.155–1.802)	0.001
BMI (kg/m^2^)	1.086 (1.067–1.106)	<0.001	1.071 (1.048–1.094)	<0.001
Height (cm)	0.983 (0.976–0.989)	<0.001		
Weight (kg)	1.010 (1.005–1.015)	<0.001		
Smoking status		<0.001		<0.001
Never	1		1	
Past smoker	1.266 (1.134–1.414)	<0.001	1.256 (1.100–1.434)	0.001
Current smoker	1.115 (0.980–1.267)	0.097	1.345 (1.154–1.568)	<0.001
Alcohol consumption		0.002		0.756
Never	1		1	
Past drinker	1.117 (0.482–2.590)	0.796	0.915 (0.362–2.310)	0.850
Current drinker	0.801 (0.707–0.908)	0.001	0.940 (0.800–1.106)	0.458
SBP (mmHg)	1.013 (1.010–1.016)	<0.001		
DBP (mmHg)	1.014 (1.010–1.018)	<0.001	1.013 (1.008–1.019)	<0.001
HbA1c (%)	1.658 (1.533–1.792)	<0.001	1.397 (1.284–1.519)	<0.001
Total cholesterol (mg/dL)	1.002 (1.000–1.003)	0.010		
LDL-C (mg/dL)	1.002 (1.000–1.003)	0.021	1.004 (1.002–1.005)	<0.001
HDL-C (mg/dL)	0.992 (0.989–0.996)	<0.001		
Triglycerides (mg/dL)^1^	1.001 (1.001–1.002)	<0.001	1.001 (1.000–1.002)	0.003
Hypertension	1.650 (1.376–1.977)	<0.001	1.566 (1.270–1.932)	<0.001
Diabetes	1.690 (1.263–2.261)	<0.001		
Dyslipidemia	1.267 (1.051–1.527)	0.013		
Ischemic heart disease	2.752 (1.045–7.247)	0.040		
Cerebrovascular disease	0.994 (0.237–4.164)	0.994		

OR, odds ratio; CI, confidence interval; BMI, body mass index; SBP, systolic blood pressure; DBP, diastolic blood pressure; LDL-C, low-density lipoprotein cholesterol; HDL-C, high-density lipoprotein cholesterol.

^1^log_e_ transformed.

## Discussion

The findings from this large longitudinal study have shown that CAC score progression was more common in individuals who had RE than in those who did not have RE at baseline. This association persisted when the model was adjusted for potential confounders that included age, sex, alcohol consumption, and the smoking status. However, the association disappeared when the model was adjusted further for BMI, DBP, the presence of hypertension, HbA1c, LDL cholesterol, and triglycerides. These findings indicate that RE could predict future CAC progression. While RE is not a direct risk factor for CAC progression, metabolic factors may play roles in CAC progression in individuals with RE.

The results from this study are consistent with those from previous studies [14,15]. Chen *et al*. [15] conducted a population-based cohort study that included 12,960 patients who had GERD and 51,840 who did not have GERD, and they showed that the risk of CHD was greater for the GERD cohort (adjusted hazard ratio, 1.49; 95% CI, 1.34–1.66; *P* < 0.001). Additionally, Shah *et al*. [14] reported that patients with GERD who had been exposed to proton pump inhibitors had a 1.16-fold increased association with myocardial infarction (95% CI, 1.09–1.24), and that this association existed regardless of the use of clopidogrel. However, these studies had important limitations, because several potential confounding factors, including BMI, WC, blood pressure, and the lipid profiles were not considered; hence, the association between GERD and CHD may have been overestimated. On the other hand, the current study incorporated detailed information about the metabolic parameters. Using high quality clinical, imaging, and laboratory procedures improved the robustness of this study’s data.

Obesity and, especially, abdominal obesity, is a strong independent risk factor for GERD [4,16]. Gastroesophageal reflux (GER) occurs when the intragastric pressure is greater than the intraesophageal pressure. An increased pressure gradient between the stomach and the esophagus is present in individuals who have high BMIs [17]. In addition to the mechanical effect of the increased pressure gradient on GER promotion, central adiposity has a BMI-independent effect on the risk of RE [18]. Recently, esophageal inflammation involving a cytokine-mediated pathway, rather than reflux, has been proposed as a mechanism that underlies the pathogenesis of RE [19]. In addition, previous studies’ findings have shown that metabolic syndrome and GERD are independently associated [16,20–23]. Chung *et al*. analyzed 7,078 adult men and women who had participated in health screening examinations, and they showed that metabolic syndrome was associated with RE (OR, 1.42; 95% CI, 1.26–1.60) after adjusting for multiple confounders [16]. Collectively, these findings suggest that we should not view RE as just a gastrointestinal problem and that it should be considered as at least one manifestation of metabolic syndrome.

Several prospective studies’ findings have shown that metabolic abnormalities and obesity are associated with an increased risk of CHD [24–29]. The findings from an observational cohort study of 19,173 participants showed that obesity and metabolic syndrome were associated with increased risks of all-cause and CHD-related mortality [26]. Similarly, the findings from two large prospective cohort studies showed that BMI and WC strongly predicted the future risk of CHD [29]. Furthermore, the findings from a recent meta-analysis of 14 prospective cohort studies demonstrated the combined effects of obesity and metabolic abnormalities on the risks of CHD and mortality [30]. In the present study, CAC progression was used as an indicator of future CHD events. Previous studies’ findings have shown that CAC progression was closely associated with metabolic syndrome [31,32].

There are some limitations to our study. First, this study was conducted on asymptomatic Korean men and women who attended regular health screening examinations, and our findings may not be generalizable to other populations. Second, only individuals who underwent EGD and repeated coronary CT scans were enrolled in this study, which may have caused a selection bias. However, given that CAC scan was optional screening test that subjects decided whether to receive, the effect of selection bias would be small. However, our study has several strengths that include its longitudinal design, large sample size, and the availability of detailed information about many metabolic parameters. In addition, the current study is the first to establish a relationship between RE and CAC progression in a longitudinal investigation.

In conclusion, RE was associated with CAC progression. However, the association disappeared after the model was adjusted for the metabolic parameters, which suggests that metabolic factors may play roles in CAC progression in individuals with RE. Our results suggest that the presence of RE could be closely associated with CHD, even though RE is not a direct risk factor associated with CHD. Thus, individuals who are diagnosed with RE should be aware of coronary atherosclerosis and try to modify the risk factors associated with CHD as well as receive acid suppressive or antireflux therapy for esophagitis.

## Supporting information

S1 File This file contains detailed information for the statistical analyses of this study. (XLS)
